# The Heart

**Published:** 1858-05

**Authors:** Horatio N. Hollifield

**Affiliations:** Sandersville, Georgia


### ARTICLE II.

*The Heart.* By Horatio N. Hollifield, A. M., M. D., Sandersville, Georgia.

This organ is one of the most vital importance to the whole animal economy. Should it by disease or by accident be caused to stop performing its functions, then life would become extinct, and man would perish.

Situated in the mediastinum between the, Lungs above the diaphragm, and between the sternum and the vertebra, it is the centre of the circulation. It is a hollow muscular organ, of a conoidal shape, the base of the cone being towards the ver. tebral column, while its apex is about the junction of the fifth rib, with its cartilage on the left side.

The average size of the heart, I find from the examination of seventeen, to be about four and three-quarter inches in length, and about three inches and a quarter in diameter at the base, and weighing five and a half ounces. It appears that it increases in size according to the age of the patient up to about fifty years. It contains four cavities, a right and left auricle, and a right and left ventricle, but are not all of them of the same capacity,because the right auricle and the right ventricle increase in size as age advances, in a much greater ratio than the left. The walls of the ventricles at birth, are near about the same thickness, but as man advances in age they go on increasing, but not in equal proportions; the walls of the right ventricle being arrested in their growth much sooner than those of the left. The walls of the right ventricle are from two to three lines in thickness, while those of the left ventricle are from five to seven lines.

From the construction of the heart, we see and know that it is a very strong and powerful organ, made so by our divine creator, in order to accomplish the great amount of work which it is its duty to perform, and in order to be able to fully appreciate its labors, it will be necessary for us to make a calculation as to the amount of work accomplished by this little organ, but one which occupies a position second to no other in the whole animal economy.

The left ventricle of the heart, in a healthy man, aged thirty-five years, I found by measurement, would contain two and a quarter ounces of blood—the heart usually empties itself about seventy-five times in a minute, so there will pass through the heart eleven thousand two hundred and fifty ounces of blood every hour, or seven hundred and one and a quarter pounds avoidupois, and in one day there will pass through the heart sixteen thousand eight hundred and thirty pounds of blood, and in one year six million one hundred and forty-two thousand nine hundred and fifty pounds; and in three-score years and ten, the duration of life allotted to man, there will pass through the heart four hundred and thirty millions and six thousand five hundred pounds of blood. In a healthy man, we have about forty pounds of blood which will pass through the heart four hundred and twenty and three-quarter times every day, and one hundred and fifty-three thousand five hundred and seventy-four times every year.

From this, many would suppose that this little organ, in order to accomplish so great a task as this which is imposed upon it, must be endowed with a power analogous to that of perpetual motion, but this is not the case, for the human heart is but a muscle, and like all other muscles of the body, it has its periods of rest, and far from being in a state of continual motion as is generally supposed, it has its alternations of action and repose, whose comparative lengthseems scarcely to differ from the proportions which many other muscles of the animal economy, and particularly the diaphragm and intercostal muscles present in this respect. In fact, in admitting by an approximate calculation approaching very near to exactness, that of the total duration of time occupied by the complete succession of the movements of the heart, one-fourth is occupied by an absolute repose of all its parts, one-half by the contractions of the ventricles, and one-fourth by the contractions of the auricles, we will find that out of twenty-four hours, the ventricles have twelve, and the auricles eighteen hours of repose. In individuals whose pulse gives habitually less than fifty pulsations a minute, the repose of the ventricles is more than sixteen hours a day.

The muscles of voluntary motion have often not more in men engaged in laborious occupations, and among those especially which serve to maintain the head and trunk in an erect position, there are some certainly that repose less, the more so as their action is not perhaps completely interrupted by sleep. (Læunec.) And although the heart does perform an almost Herculean task, yet it is not always at work, but nature allows it in common with all other created things, a period of repose, so that it can rest from its labors, and be enabled to recruit its strength and be capable and ready always to perform its proper functions with regularity and precision.

